# Allele co-segregation and haplotype diversity of MHC IIβ genes in the small-spotted catshark *Scyliorhinus canicula*

**DOI:** 10.1007/s00251-025-01376-w

**Published:** 2025-03-31

**Authors:** Alessia Rota, Ana Veríssimo, Arnaud Gaigher

**Affiliations:** 1 Department of Earth and Environmental Sciences, University of Milano-Bicocca, Milan, Italy; 2 National Biodiversity Future Centre (NBFC), 90133 Palermo, Italy; 3 CIBIO, Centro de Investigação em Biodiversidade e Recursos Genéticos, InBIO Laboratório Associado, Campus de Vairão, Universidade do Porto, 4485-661 Vairão, Portugal; 4 BIOPOLIS Program in Genomics, Biodiversity and Land Planning, CIBIO, Campus de Vairão, 4485-661 Vairão, Portugal; 5 Research Unit for Evolutionary Immunogenomics, Department of Biology, University of Hamburg, Hamburg, Germany

**Keywords:** Major histocompatibility complex, Shark, Allelic lineages

## Abstract

The major histocompatibility complex (MHC) constitutes a functionally relevant multigene family playing an essential role in the adaptive immune responses of jawed vertebrates, being directly involved in pathogen recognition. MHC diversity, driven by pathogen-mediated selection, is vital for species survival and is characterized by high genetic diversity in many taxa, namely at the sequence, allelic and haplotype levels. Chondrichthyans, the most basal jawed vertebrates with an adaptive immune system, exhibit a high diversity of MHC gene lineages conservatively organized in a compact region of the genome. Such genomic architecture suggests linkage among MHC genes, where alleles from different genes possibly co-segregate together. Such condition may have major implications on immune response, individual fitness and evolution. In this study, we examine MHC IIβ haplotype diversity in a model shark species, the small spotted catshark, *Scyliorhinus canicula.* Making use of pedigree data, we reconstructed MHC IIβ haplotypes to understand allele transmission from parent to offspring. Results indicate allele co-segregation consistent with tight linkage among MHC IIβ genes, suggesting the presence of functional stable haplotypes inherited from parents to offspring. The reconstructed haplotypes suggested extensive haplotype diversity characterized by variable allele numbers and allelic lineage composition, as well as marked allelic divergence, consistent with previous population-level data on this species. These findings underscore the complexity of MHC genetics (and of MHC evolution) in chondrichthyans. Accurate reconstruction of MHC haplotypes and assessment of its functional significance are crucial for better understanding adaptive immune responses and MHC evolutionary dynamics in chondrichthyans.

**Supplementary Information:**

The online version contains supplementary material available at 10.1007/s00251-025-01376-w.

## Introduction

The major histocompatibility complex (MHC) is a large cluster of genes found across all jawed vertebrates, which code for transmembrane glycoproteins. MHC proteins are responsible for antigen binding and presentation to T-cells derived from intracellular (e.g. virus; MHC class I genes, MHC I) and extracellular pathogens (e.g. bacteria, eukaryote parasites; MHC class II genes, MHC II), thereby eliciting a cascade of reactions aimed at preventing infection and disease (Neefjes et al. 2011). These proteins are thus essential players in immune responses and are among the main hallmarks of the adaptive immune system, in addition to T-cell receptors and immunoglobulins.

Genetic diversity at MHC genes is an essential element in the long-term survival of any species, and it is often fine-tuned to the host’s pathogen community. As a result of the host–pathogen arms race (via pathogen-mediated selection), MHC genes have evolved the highest level of genetic polymorphism known in vertebrates (e.g. humans exhibit 1000s of MHC alleles; Robinson et al. 2019). This diversity has been shown to directly correlate with pathogen resistance, reproductive success and survival (e.g. Bateson et al. 2016; Phillips et al. 2018; Huang et al. 2022; Schmid et al. 2023; Ferreira et al. 2024). In addition to high allelic diversity and high sequence divergence between alleles, another hallmark of MHC diversity is the occurrence of copy number variation (CNV). Indeed, the number and genomic organization of MHC genes can vary greatly not only among species (Kelley et al. 2005; O’Connor et al. 2016; He et al. 2021; Minias et al. 2021; Westerdahl et al. 2022; Heimeier et al. 2024), but also among individuals of the same species (e.g. Gaigher et al. 2016; Biedrzycka et al. 2017; Wong et al. 2022).

Chondrichthyans or cartilaginous fish (chimaeras, rays and sharks) are the most basal jawed vertebrates to exhibit a mammalian-like adaptive immune system, including the MHC, T-cell receptors and immunoglobulins. Despite their ancient origin (> 450 Myr) and basal phylogenetic position, chondrichthyans exhibit a remarkable diversity of MHC class I lineages, with one lineage of classical class I (MHC Ia) genes and at least six additional lineages of non-classical class I genes of (yet) unknown functions (Wang et al. 2003; Almeida et al. 2020a, 2021). In turn, chondrichthyan MHC class II alpha and beta genes (MHC IIα and MHC IIβ) exhibit one to two lineages (e.g. DAA/DBA and DAB/DBB, depending of taxon), ranging from two to three copies each (at least), while non-classical MHC class II genes are apparently absent in the group (Kasahara et al. 1993; Dijkstra et al. 2013; Almeida et al. 2020b). At the population scale, high MHC polymorphism in chondrichthyan taxa was suggested by the first studies conducted (Kasahara et al. 1993; Okamura et al. 1997). This pattern was recently confirmed using Illumina sequencing in a model shark species, the small spotted catshark (*Scyliorhinus canicula*), and showed levels of sequence and allelic diversity at MHC IIβ genes to be as high or even higher than that reported in mammals or birds (Gaigher et al. 2023).

Interestingly, MHC class I and II genes are closely located in the genomes of elasmobranchs (sharks and rays), showing a primordial linkage between the classical class I region (i.e. “class Ia region”, including antigen processing genes) and the class II region (including only MHC IIα and MHC IIβ genes) (Ohta et al. 2000; Flajnik and Kasahara 2001; Veríssimo et al. 2023). Such compact genomic architecture of the elasmobranch MHC raises the hypothesis of tight linkage among the genes within each of the class Ia and II regions, whereby genes may co-segregate together instead of independently. This condition may have important consequences to the evolution and diversity of MHC genes in this taxonomic group, possibly resulting in the generation of superhaplotypes that are inherited as a block and allow for tight co-evolution among genes in each block. This condition has been previously shown in chicken with the tight co-evolution of MHC I and TAP variants (Kaufman et al. 1999; Walker et al. 2011).

Following up on our previous work aiming at clarifying the workings of the MHC system in elasmobranchs, here we explore haplotype diversity in MHC class IIβ genes regarding the variability in allele composition and number, and the presence of allele co-segregation among genes, using the small spotted catshark *Scyliorhinus canicula* as a case study. This species has three duplicated MHC IIβ genes within the same genomic region spanning ~ 1 Mbp and including MHC IIα genes (based on the reference sScyCan1.1 genome, assembly accession GCF_902713615.1). Previous work showed the presence of high sequence diversity at the exon 2 among MHC IIβ alleles, which formed three distinct clusters (referred as allelic lineages A to C) based on phylogenetic analysis (Gaigher et al. 2023). Each of the MHC IIβ lineages at exon 2 was represented by a single gene in the reference genome of *S. canicula* (Gaigher et al. 2023). It was further showed that *S. canicula* MHC IIβ genes have high allelic diversity overall, with each individual varying in the total number of alleles (three to six; i.e. consistent with at least three gene copies) (Gaigher et al. 2023). In the present study, we take advantage of pedigree (family) data to reconstruct MHC IIβ haplotypes to infer how MHC alleles are transmitted from parents to offspring and specifically if MHC IIβ loci are segregating independently. We further explore available genomic resources and population-level data on MHC IIβ allelic diversity from *S. canicula* (in Gaigher et al. 2023) to gain a better understanding of the genetic architecture of MHC IIβ genes. This information is also crucial to the accurate screening of MHC IIβ genes in elasmobranchs in future studies using these genes to explore other types of questions, such as mating systems and sexual selection, host–pathogen dynamics or genetic population structure.

## Material and methods

### Sampling and DNA extraction

We used two groups of *S. canicula* adult individuals and their progeny kept in aquaria under controlled conditions. The groups differed in source population and number of putative parents and offspring as follows: (1) group 1 included a single mother captured in the wild off Lisbon, Portugal (western Iberian waters), and her 21 offspring, and was held at the Laboratório Marítimo da Guia — University of Lisbon (the number of fathers was unknown); (2) group 2 included seven randomly mating adult individuals (3 fathers and 4 mothers) and their 47 offspring and was held at the Ozeaneum, Stralsund, Germany. Fin clips were taken from each individual and stored in 98% ethanol at − 20 °C, until DNA extraction and processing. Genomic DNA (gDNA) was extracted using the Molecular Biology EZ-10 Spin Column genomic DNA minipreps Kit (Bio Basic Inc.) for group 1 samples, whereas group 2 samples were provided as gDNA extractions performed with the Isolate II Blood and Tissue kit (Bioline).

### Microsatellite genotyping and family reconstruction

To ascertain the parent–offspring relationships and reconstruct the respective families, all individuals from the two groups of parent–offspring samples were genotyped at 11 microsatellite loci as described by Griffiths et al. (2011). The microsatellite loci were amplified through a single multiplex polymerase chain reaction in a final volume of 10 μl, including: 1 μl of primer mix (Table S1), 1 μl gDNA, 3 μl of autoclaved distilled water and 5 μl of Multiplex Master mix Qiagen (containing the HotStarTaq DNA Polymerase and the multiplex PCR buffer; Qiagen NV, Venlo). The thermocycling conditions consisted of an initial denaturation step at 95 °C for 15 min, followed by 35 cycles of denaturation at 94 °C for 30 s, annealing at 60 °C for 90 s and elongation at 72 °C for 45 s and a final extension step at 72 °C for 30 s.

Family reconstruction and parentage analyses were performed for group 2 individuals using Cervus (3.0 version by Field Genetics Ltd) which calculates allele frequencies for a given set of microsatellite loci directly from the genotyped individuals and subsequently simulates genotypes and calculates their likelihood ratios (using the likelihood equations of Kalinowski et al. 2007). The likelihood ratios are usually expressed as LOD scores (natural log of the likelihood ratios). Analyses were performed to assess maternity, paternity and parent-pair relationships for all the offspring and to infer the number of offspring for each parent independently and for each parental pair. All the relationships that were consistent across independent analyses and with a positive LOD score were considered as valid.

### MHC IIβ amplification, library preparation and Illumina sequencing

Genotyping of the MHC IIβ genes was performed for all reconstructed families of *S. canicula* (as described above) using the two-step PCR amplification, library preparation and sequencing protocols described in Gaigher et al. (2023). Briefly, amplification of the exon 2 (β1 domain) of MHC IIβ loci was performed using two primer pairs: NF2-NR2 was used to co-amplify alleles from lineages A and B, and DF2-DR2 was used to amplify lineage C alleles (Table S2). The resulting amplicons were cleaned with AMPure XP Beads (0.97x) (Beckman Coulter™ Agencourt) prior to the second PCR step, which adds unique barcodes (MK indexes, Meyer and Kircher 2010; Kircher et al. 2012) and Illumina adapters to each cleaned amplicon. The indexed PCR amplicons were cleaned again using AMPure XP Beads (0.8 ×) and checked for quality on 2% agarose gel electrophoresis and for quantity using the BioTek Epoch microplate spectrophotometer (Agilent Technologies, Inc.). Samples were pooled equimolar into primer pair-specific libraries (i.e. NF2-NR2 and DF2-DR2 libraries) and normalized to 20 nM. The libraries were tested for quality, concentration, size and integrity using the 2200 TapeStation System (Agilent Technologies Inc., Santa Clara, USA) and validated using the KAPA Library Quantification Kit (KAPA Biosystem, Inc., Wilmington, USA) for Illumina sequencing platforms, following the manufacturer’s protocol. The two libraries were combined using a 2:1 ratio for NF2-NR2:DF2-DR2, in order to have proportional read coverage between lineages A and B (NF2-NR2) and lineage C (DF2-DR2) alleles. The final library was sequenced with a MiSeq Reagent Kit v2 250PE at the Centre for Molecular Analysis in CIBIO-InBIO (Vairão, Porto, Portugal), using 20% PhiX. Reliability of the sequencing was evaluated by including 20 sample replicates, in addition to PCR blanks.

### Illumina data processing and MHC genotyping

The detailed filtering protocol can be found in Gaigher et al. (2023). Briefly, raw reads were demultiplexed using a custom made Perl script and saved as FASTQ files. Quality and size filtering of reads as well as adaptor trimming were performed with Cutadapt (Martin 2011). Filtered reads were further processed with DADA2 pipeline (Callahan et al. 2016) as follows: (i) primer trimming from forward and reverse reads, (ii) dereplication of identical reads into unique sequences, (iii) merging paired reads based on full agreement in the overlapping region, (iv) removal of potential chimeric sequences and (v) extraction of the final amplicon sequence variant (ASV) table. Further filtering of the retrieved ASVs was performed to reduce the number of artefacts. First, samples with a final coverage < 100 sequences and variants with a maximum coverage < 10 were removed from the dataset. Second, the ASVs were aligned in Geneious Prime v22.1, and variants differing from the targeted loci were discarded. Third, variants with frequencies < 1% (per-amplicon) were automatically considered as artefacts and removed. As artefacts at higher frequency may still remain in the final ASV table, the final classification of variants as artefacts was based on the following assumptions: (i) variants should amplify similarly across samples (but see Sommer et al. 2013), (ii) artefacts should be less frequent than true alleles and (iii) chimeric sequences or sequences with single base pair mismatches should co-occur with their parent sequences in the same sample (Sommer et al. 2013; Lighten et al. 2014; Biedrzycka et al. 2017; Rekdal et al. 2018; Gaigher et al. 2018). Therefore, variants with low frequencies that are found at higher frequencies in other amplicons (defined as true variants/alleles) were treated as artefacts (Gaigher et al. 2023). Chimeras and single base pair substitutions compared to true alleles were removed (Gaigher et al. 2023). Once the final sample dataset and the putative true alleles have been defined, we proceeded to lineage attribution for the co-amplified MHC IIβ-A and MHC IIβ-B alleles based on diagnostic nucleotide sites and phylogenetic networks of β1 (exon 2) and β2 (exon 3) (Gaigher et al. 2023). Allele assignment is therefore performed at the lineage-level only.

### Assessment of allele co-segregation and haplotype diversity

We used the pattern of allelic segregation within families to reconstruct MHC IIβ haplotypes. The offspring’ haplotypes were inferred assuming that the maximum number of alleles can differ between individuals (as showed in Gaigher et al. 2023). In addition, due to the occurrence of multiple paternity and the potential for sperm storage in *S. canicula* (Griffiths et al. 2012), offspring sired by the same mother may present alleles deriving from different putative fathers. Due to the specific mating system of *S. canicula*, families were defined by the mothers.

From the resulting haplotypes, we investigated the hypothesis of allele co-segregation (linkage) among MHC IIβ lineages in *S. canicula* given their physical proximity in the genome (Gaigher et al. 2023). For instance, assuming four different alleles (two loci) in each heterozygote parent, we expect to observe a maximum of 16 different haplotypes in the offspring if alleles are segregating independently. However, we expect a maximum of only four different haplotypes in the offspring if two loci are tightly linked. Following this rationale, we deduced the frequency of recombinant haplotypes in our family data, which reflects the linkage between loci. To avoid any bias in detecting recombination events, only parents with different MHC IIβ allelic compositions and with a minimum of five offspring were considered for analysis. The condition of parents with only different alleles was applied only to MHC IIβ-C, as the lineage-specific amplification automatically results in 100% allelic coverage in the offspring if the parents have a unique and similar allele (the offspring can have only one or two identical alleles, the coverage remains the same).

High levels of polymorphism within the MHC region can result in allele- or locus-bias amplification when using a unique pair of primers to co-amplify two or more loci. Consequently, a missing allele or locus can be due to a methodological bias; thus*,* new lineage-specific primer pairs were designed to confirm allele identity and exclude possible bias amplification of the original primer pairs. Specifically, five new primer pairs were designed targeting both the β1 (exon 2) and the β2 (exon 3) domains of the MHC IIβ genes; Fig. S1; Table S2). Each exon was amplified using a master mix with a 5-µl total volume, including 2.5 µl of MyTaq HS Master mix, 1.5 µl of autoclaved water, 0.2 µl of each primer (10 µM) and 0.6 µl of the gDNA. The thermocycling conditions consisted in an initial denaturation step at 95 °C for 3 min, followed by 35 cycles of denaturation at 95 °C for 30 s, annealing at each primer-pair Ta (Table S2) for 30 s, extension at 72 °C for 30 s and a final extension step at 60 °C for 10 min. The obtained amplicons were cleaned of excess primers and dNTPs with 1 µl of ExoSap-IT™ (Thermo Fisher) and processed for Sanger sequencing of the forward and reverse strands using the BigDye™ Terminator v3.1 Cycle Sequencing Kit following the manufacturer’s instructions. All products were sequenced on an ABI Prism 3130 Genetic Analyzer. The final Sanger sequences were manually inspected and edited in Geneious Prime and aligned to reference alleles attributed to each MHC IIβ lineage (A, B and C; Gaigher et al. 2023). The allele and lineage presence and identities were confirmed when different primers targeting the same exon yield similar results.

Genetic diversity at the sequence and allelic levels within each of the reconstructed MHC IIβ haplotypes was calculated using different metrics: (i) nucleotide p-distances, (ii) amino acid p-distances and (iii) amino acid functional distances. While p-distances between alleles within haplotypes were estimated with MEGA11 (Tamura et al. 2021), the functional distance was calculated with Grantham's distance considering the physicochemical properties of the respective amino acids using a Perl script from Pierini and Lenz (2018).

### Genetic architecture of MHC IIβ

To gain further insights into the genetic architecture of MHC IIβ allelic lineages in *S. canicula*, and based on observations from the reference genome (sScyCan1.1, GenBank accession no. GCF_902713615.1), we explored the hypothesis that alleles in each allelic lineage were derived from distinct MHC IIβ loci. Specifically, we expect that allelic lineages derived from distinct loci should exhibit divergent untranslated regions (UTR; e.g. Okamura et al. 1997). To test for this, full transcripts from the three MHC IIβ genes in the reference genome, belonging to each of the three allelic lineages, were obtained from NCBI (Accession no.s XR_005462827, XM_038815839 and XM_038816327, for lineages A, B and C, respectively), and their 5′ and 3′ UTR regions were aligned in Geneious Prime® 2023.0.1 using the MUSCLE algorithm. Also, if allelic lineages segregate in distinct loci, the number of alleles per lineage per individual should never exceed two and should be independent between lineages. These conditions were assessed using data on the allele composition at MHC IIβ genes for 25 unrelated individuals of *S. canicula* obtained in Gaigher et al. (2023).

## Results

### Family reconstruction using microsatellite loci

Multilocus microsatellite genotypes were obtained for all sampled individuals, except for a single offspring from group 1 which failed amplification (Table S3). The microsatellite genotype data of group 1 revealed the presence of a minimum of three unsampled putative fathers, based on the number of alleles per locus not detected in the mother (two to six alleles). Family reconstruction of group 2 individuals resulted in 55% of the offspring being successfully assigned to a sampled parent pair (*n* = 26; Table S4), while the remaining did not obtain a robust paternal or maternal assignment (Table S4). As mentioned earlier, given the presence of multiple paternity in *S. canicula* (Griffiths et al. 2012), a family was considered solely based on the mother and her offspring. Thus, our data comprised a total of five families hereon referred to as family #1 (group 1) and families #2 to #5 (group 2).

### MHC IIβ genotyping

Of the 76 individuals sampled, 64 were successfully genotyped for MHC IIβ-A/B and 44 for MHC IIβ-C, with an average coverage per individual of 4683 and 2835 sequences, respectively. The discrepancy in genotyped individuals between MHC IIβ-A/B and MHC IIβ-C was due to no amplification of the latter in 26 individuals (see below for details). Twelve individuals (six from group 1 and six from group 2) were excluded during the raw data cleaning and filtering steps. Upon sequence filtering, we retained a total of 29 variants considered as true alleles. Based on sequence diversity and phylogenetic networks, five alleles were attributed to MHC IIβ-A, 20 to MHC IIβ-B and four to MHC IIβ-C (Figs. S2 and S3). Out of the 29 alleles recovered, 20 were previously reported in Gaigher et al. (2023) and the nine new alleles were submitted to GenBank (Accession numbers: PP982215-PP982223). All sample replicates for both primer pairs were 100% congruent in the detected alleles. The MHC IIβ dataset including alleles from all lineages (A, B and C) consisted of 64 individuals in total, namely group 1: 15 offspring and 1 mother, and group 2: 41 offspring and 7 putative parents.

The validation of the number of MHC IIβ lineages and the allele identity per MHC IIβ lineage obtained with Illumina sequencing was performed with lineage- and domain-specific amplification and Sanger sequencing. Full congruence was found for all samples at the lineage and allele levels except for two offspring samples from group 2, which produced no data from Sanger sequencing and were removed from the dataset. On the other hand, Sanger sequencing of β1 domains produced new allelic sequences for a total of five offspring samples (four offspring for group 1; one offspring for group 2) that failed/were filtered out during Illumina sequencing and processing. Data for these individuals were added to the MHC IIβ dataset produced by Illumina sequencing (Table 1).

**Table 1:** Summary of sample sizes per molecular marker and sequencing approach for each group of *Scyliorhinus canicula* individuals. Adult individuals are shown as M for mothers and F for fathers

	Microsatellite loci	MHC IIβ — Illumina	MHC IIβ — Sanger
Group 1	1 M + 21 offspring	1 M + 15 offspring	1 M + 19 offspring
Group 2	4 M + 3F + 47 offspring	4 M + 3F + 41 offspring	4 M + 3F + 40 offspring
Total	76	64	67

Congruence of MHC IIβ alleles detected in the offspring and in the reconstructed parental pair was assessed for group 2 to evaluate consistency between MHC IIβ and microsatellites. Full congruence between offspring and parental pair MHC IIβ alleles was obtained for 87% of the offspring with confident parental assignment (*n* = 20), while the detected MHC IIβ allelic composition was fully congruent with proposed parents in 77% of the offspring without confident parental assignment (*n* = 13). The discordances were due to sample mislabelling or to alternative parental assignment (*n* = 1) as detailed next. Six offspring had MHC alleles discordant to those of the reconstructed parent pair. In these cases, sample mislabelling during microsatellite genotyping was assumed given (a) full congruence between Illumina and Sanger sequencing for each sample and (b) the extensive lineage and domain validation steps taken to confirm number and identity of MHC IIβ alleles for all three lineages, i.e. using different primer pairs for the same samples. Therefore, family reconstructions for the mislabelled offspring were based on the MHC genotypes, and the samples were kept for further analysis. One offspring had MHC alleles incompatible with those of the assigned father at one microsatellite locus, while the alternative father based on MHC alleles was fully compatible at the microsatellite all loci. In this case, the alternative parent–offspring pair suggested by MHC alleles was considered for further analysis. The final MHC IIβ dataset used in assessing allele co-segregation and haplotype diversity included 67 individuals (Table 1).

### Allele co-segregation and haplotype diversity in MHC IIβ

Family data showed evidence of allele co-segregation among the investigated MHC IIβ lineages. Allele co-segregation between the MHC IIβ A, B and C lineages is evident in the presence of only two haplotypes transmitted by each parent to the offspring, e.g. Family #5 — mother 4 with two haplotypes: DBB*17(B)/DBB*11(A) and DBB*06(A)/DBB*55(C); father 1 with two haplotypes: DBB*35(B)/DBB*41(B) and DBB*03(A)/DBB*16(B); and father 3 with DBB*68(B)/DBB*69(B) and DBB*67(B)/DBB*45(C) (Tables 2 and 3). This observation is consistent across all families (Table S5). Our family dataset revealed a total of 14 different haplotypes, with distinct lineage and allelic compositions (Table 3). All parental haplotypes consist of maximum 2 alleles, with the exception of haplotype H8, which presents only a single allele (Table 3), consistent with the presence of (at least) two MHC IIβ genes. Among the described haplotypes, three are composed of both A and B alleles, two of A and C alleles, three of B and C alleles and six haplotypes show only B alleles (Table 3). No recombination event was detected among MHC IIβ lineages based on the haplotype reconstruction of each offspring in the dataset (Table 3).

**Table 2:** Haplotype reconstruction of MHC IIβ lineages in *S. canicula *showing allele co-segregation using family data. This example illustrates the Family #5 (father 2 is not included as he contributed to only one offspring in this family). Grey and white shading indicate the presence and absence of alleles in offspring, respectively. All allele names (i.e. Scca-DBB*06) were reduced to the allele number. Letters A, B and C refer to the three different MHC IIβ lineages

		Mother 4 alleles				Father 1 alleles					Father 3 alleles			
		A and B			C	A and B				C	A and B			C
		17	11	06	55	35	41	03	16	-	67	68	69	45
Offsprings´ alleles	1G2	B	A			B	B							
	1B2	B	A					A	B					
	1C4			A	C			A	B					
	1H5			A	C			A	B					
	2D1			A	C			A	B					
	1J3			A	C	B	B							
	1B6			A	C							B	B	
	2C1			A	C							B	B	
	2B9			A	C							B	B	
	1A3			A	C						B			C
	1I10	B	A								B			C
	1J1	B	A								B			C

**Table 3:** MHC IIβ haplotypes of *Scyliorhinus canicula* inferred from allele segregation in five families. Each Scca-DBB allele is associated with a specific lineage (MHC IIβ-A, MHC IIβ-B or MHC IIβ-C), and all allele names were reduced to the allele number. Fathers with * indicate that although their full MHC allele composition is unknown, their specific haplotypes were observed in at least five offspring. Mother 2 and father 2 were not included as they have less than five offspring. Three genetic distances are represented: (i) the nucleotide p-distance (Nucl p-dist), (ii) amino acid p-distance (AA p-dist) and (iii) amino acid functional distance (Func dist)

Haplotype	Group	Family	Origin	Offspring	Scca-DBB alleles				Nucl p-dist	AA p-dist	Func dist
					A	B	B	C			
H1	1	#1	Mother	12		17	71		0.143	0.238	15.39
H2	1	#1	Mother	7		31		50	0.298	0.418	28.49
H3	1	#1	Father*	5		21	70		0.164	0.338	21.26
H4	1	#1	Father*	6	65			51	0.324	0.481	36.43
H5	2	#2	Mother 1	5		42		45	0.366	0.544	36.32
H6	2	#2	Mother 1	6	03	21			0.193	0.350	23.68
H7	2	#4	Mother 3	1		18	33		0.107	0.213	16.25
H8	2	#4	Mother 3	5		72			NA	NA	NA
H9	2	#5	Mother 4	4	11	17			0.168	0.288	18.74
H10	2	#5	Mother 4	9	06			55	0.336	0.519	37.13
H11	2	#2, #4, #5	Father 1	14		35	41		0.127	0.238	17.59
H12	2	#2, #3, #4, #5	Father 1	9	03	16			0.193	0.350	23.01
H13	2	#2, #5	Father 3	5		68	69		0.135	0.225	17.19
H14	2	#5	Father 3	3		67		45	0.311	0.456	28.90

The within-haplotype diversity values of *S. canicula* adults were strongly correlated among the three genetic distances used (*r*^2^ > 0.93) and varied depending on which lineages were present. Specifically, all haplotypes carrying lineage C alleles (in addition to A or B alleles) showed higher among-allele genetic distances and functional divergence, while the haplotypes with only B lineage alleles presented the lowest values (Table 3). Those results are in line with the observed sequence divergence among alleles from distinct lineages, as shown in Figures S2 and S3.

### Genetic architecture of MHC IIβ

Marked divergence of the 3′ UTR region was found in the lineage C allele compared to lineage A and B alleles (44–45%), with alleles from lineage A and B showing high similarity (98%; Fig. S4). These observations suggest that lineage C alleles derive from a distinct locus. These results are consistent with those obtained based on population-level data on MHC IIβ genes from Gaigher et al. (2023). Specifically, the number of alleles per lineage per individual did not exceed 2 for lineages A and C; however, it varied from 1 to 4 for lineage B (Table S6). No relationship was found between the number of lineage C alleles (1–2) and the number of lineage A or B alleles per individual (Fig. S5a, b). In contrast, the number of lineage A alleles (0 to 2) decreased with the number of lineage B alleles (1 to 4), and the sum of A and B alleles never exceeded 4 (Fig. S5c). Taken together, these observations suggest that allelic lineages A and B are segregating in the same loci.

**Fig. 1:**
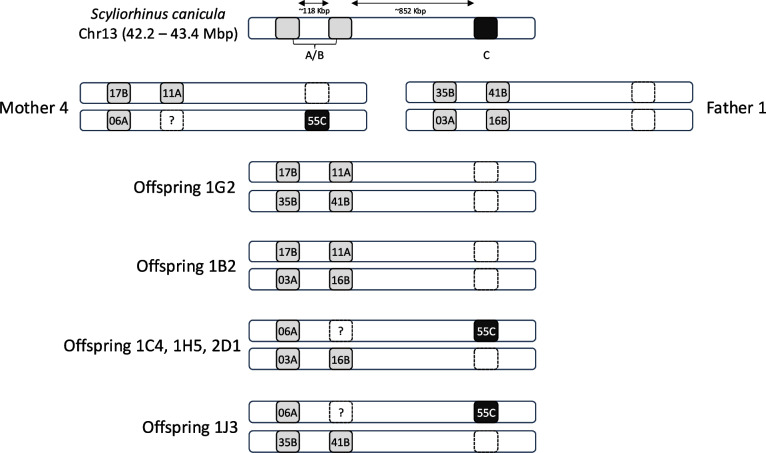
Genetic architecture of MHC IIβ in *S. canicula* and the hypothesized hemizygous condition for the lineage C gene. Haplotypes are derived from the parental pair and offspring of family #5. Lineage A and B alleles are shared by the same loci (depicted in light grey), and lineage C is encoded in a separate locus (depicted in black). The location of the genes in chromosome 13 is based on the reference genome (sScyCan1.1, GenBank accession no. GCF_902713615.1), but the location of lineage A/B alleles in the haplotypes is hypothetical. Question mark — may be a true absence or a homozygous condition for allele 06A

Based on the above finding, the reconstructed haplotypes from family data allow for an alternative interpretation with regard to the number of gene copies per haplotype, in particular for the lineage C locus (Fig. 1). First, 34% of all the offspring had no allele for lineage C (e.g. sample 1G2 in Table 2 and Table S5), suggesting the absence of this gene in the genome. Second, some parents exhibiting a single lineage C allele did not transmit that allele to all their offspring, as expected in homozygous individuals (e.g. gene C in family #2 offspring 1I1; Table 2 and Table S5). This observation suggests instead a hemizygous condition (i.e. the gene is present on only one chromosome in a homologous pair) in which the parents are heterozygotes for presence/absence of that gene copy (Fig. 1).

## Discussion

In the present study, we took advantage of family- and population-level data to explore the haplotypic diversity and genetic architecture of MHC-IIβ genes of a model shark species, the small-spotted catshark *S. canicula*. The current genotyping protocol was validated by the coherence of individual allelic compositions within families and consequently considered robust for our downstream analyses.

Our family reconstructions coupled to our MHC genotyping protocol showed that alleles at the three MHC IIβ lineages in *S. canicula* were consistently transmitted from the parents to the offspring in a maximum of two composite haplotypes. Thus, the linkage between the MHC IIβ loci is strong in this shark species. This result is in line with expectations based on the genomic organisation of MHC IIβ genes reported for *S. canicula*, where all genes are physically located within the same genomic region encompassing ~ 1 Mbp and also including MHC IIα genes (Chr. 13 from the reference genome sScyCan1.1) (Gaigher et al. 2023). Our results thus raise the hypothesis that the observed linkage pattern may comprise both MHC class IIα and IIβ loci, possibly extending into the neighbouring “MHC region”. Indeed, MHC class I and class II genes, together with several MHC-related syntenic genes, are closely located to each other in elasmobranchs (sharks and rays) and form a compact “MHC region” (Veríssimo et al. 2023). Such genomic architecture would favour the non-random association of alleles from several immune-related loci, possibly leading to major functional and ecological advantages in cartilaginous fish populations.

The tight linkage among MHC and MHC-related genes is a common feature in jawed vertebrates and has been previously reported in tetrapods and teleosts (e.g. Nonaka et al. 1997; Flajnik et al. 1999; Kaufman et al. 1999; Stenzel et al. 2004; Tsukamoto et al. 2009; McCConnell et al. 2014; Gaigher et al. 2016, 2018), as well as in other sharks (Ohta et al. 2000; Zhang et al. 2020; Almeida et al. 2020b). Co-segregation of MHC alleles with co-adapted alleles from other linked immune-related genes into stable haplotypes has been previously described in the chicken (Walker et al. 2011). Such stable haplotypes were shown to be involved in disease resistance (Kaufman et al. 1999; Wallny et al. 2006). The screening of the entire MHC region in elasmobranchs using long-read sequencing and family data could assess the extension and gene composition of the MHC haplotypes, opening the way to follow-up studies linking specific haplotypes to disease resistance or fitness-related traits.

Our study also showed extensive haplotype variation in MHC IIβ genes in the small-spotted catshark characterized by variable allele number and lineage composition. Our results further suggest that allelic lineage C of *S. canicula* MHC IIβ segregates in a distinct locus from lineages A and B. The absence of MHC IIβ-C alleles in the offspring was observed in cases where each reconstructed parent showed one allele for that gene (e.g. in family #2 offspring 1I1; Table S5). The above observation implies that each parent may be heterozygous for the presence of the MHC IIβ genes, i.e. each gene may occur in a hemizygous condition in which one DNA strand transmits the gene while the other does not (Fig. 1). A similar condition may also occur for lineage A and B, but the current samples and data do not allow for robust testing of this hypothesis. However, based on the number of combined lineage A and B alleles per individual in the population dataset (one to four), the number of loci may vary between one and two.

Variability in allele number among individuals may result from some alleles being undetected because of biased amplification across alleles. Such issues have been previously reported in MHC studies and can lead to incomplete isolation of MHC genes and diversity (e.g. Sommer et al. 2013; Burri et al. 2014; Marmesat et al. 2016). To exclude this possibility, further sequencing has been carried out in the studied families and for all three MHC IIβ lineages independently, by targeting both MHC IIβ exon 2 (with different primer pair) and exon 3. Complete agreement among primer pairs has been observed regardless of lineage.

It can be hypothesized that different MHC II allelic combinations, in terms of number of alleles and nucleotide diversity/divergence among alleles, may result in MHC II proteins interacting with distinct antigens. For instance, the MHC IIβ-C gene is highly divergent at amino acid residues expected to interact with antigen peptides when compared to MHC IIβ-A and MHC IIβ-B lineages (Gaigher et al. 2023). Indeed, individuals carrying haplotypes with lineage C alleles showed higher functional divergence compared to haplotypes with lineage A and/or B alleles (Table 3). Consequently, the resulting proteins may perform differently from proteins containing IIβ-A or IIβ-B. On the other hand, the number of functional MHC genes in an individual can be limited by the effect of the high number of MHC molecules on the reduction of the self-reacting T-cell repertoire (Nowak et al. 1992; Kasahara et al. 1993; Migalska et al. 2019). Therefore, the expansion of the MHC gene family can be costly in terms of efficiency of the immune response. In this context, the presence of only one copy of an MHC gene in hemizygous individuals may be beneficial in reducing the number of functional alleles while preserving the advantages of having one more MHC molecule (Wong et al. 2022). Further insights on haplotype diversity and the optimal number of functional MHC genes in elasmobranchs could be relevant to improve our understanding of the population fitness and the mechanisms involved in shaping MHC gene family evolution.

Some considerations should be done regarding possible limitations of our dataset. To begin with, inferring robust allele co-segregation patterns may be difficult in cases where MHC allele composition is unknown for one of the parents (e.g. group 1 — family #1, at least three unsampled fathers) or when the number of offspring for a given parental pair is small (e.g. group 2 — mother 2 sired only two offspring). Although our study reveals consistent patterns of allele co-segregation in all families of *S. canicula* surveyed, it still relies on a small sample size. Aiming at larger sample sizes in the number of families and the number of offspring will not only improve the statistical power (Gaigher et al. 2019) but also allow to estimate the haplotype frequencies to test whether a specific MHC haplotype composition is selectively advantageous (e.g. Gaigher et al. 2018) or test the fitness consequences of common or rare MHC haplotypes in natural populations (e.g. Phillips et al. 2018). Additionally, when performing phylogenetic clustering, often high supported sequence clusters are recognized as alleles stemming from the same locus. However, such interpretations may be erroneous due to the complex evolutionary dynamic of the MHC system, i.e. a specific locus can retain highly divergent MHC alleles (e.g. Tsukamoto et al. 2009) while highly similar alleles can be shared between loci due to homogenization by gene conversion. Lastly, the reconstruction of the full composite MHC IIβ haplotype requires independent validation. Coupling short-read amplicon sequencing with the long-read sequencing technology will considerably improve the reconstruction of the structural genetic variation of MHC (e.g. O’Connor et al. 2019; He et al. 2021; Westerdahl et al. 2022; Mellinger et al. 2023) and will eventually confirm the detected haplotype diversity. This approach will also help clarify the inherited composite haplotypes in cases where the parents of a given offspring share the same allele.

## Concluding remarks

Here, we show evidence of allele co-segregation and extensive haplotype diversity in MHC IIβ genes in a model shark species, making use of family and population-level data and Illumina high throughput sequencing. Our results suggest that the MHC II region of elasmobranchs (including alpha and beta genes) may be inherited as a block, and it raises the hypothesis that such linkage may extend to the adjacent MHC class Ia region. Variability in allelic compositions among haplotypes of *S. canicula* was found at genes known to have divergent peptide binding residues, possibly maximizing functional allelic diversity at the individual-level. Follow-up studies using a larger number of families and litter sizes coupled to long read sequencing will be instrumental in clearly defining the composition and extension of MHC haplotypes, their evolutionary history and ecological implication.

## Supplementary Information

Below is the link to the electronic supplementary material.

The online version contains supplementary material available at (DOCX 359 KB). 

## Data Availability

Previously identified MHC IIβ sequences are available in GenBank (accession numbers: OQ123732-OQ123795). MHC IIβ sequences described in this study were deposited in GenBank (accession numbers: PP982215-PP982223).
