# Supplementary material for: Allele co-segregation and haplotype diversity of MHC IIβ genes in the small-spotted catshark Scyliorhinus canicula
Source: Immunogenetics. 2025 Mar 31;77(1):19. doi: 10.1007/s00251-025-01376-w (PMC11958417; doi:10.1007/s00251-025-01376-w)
Supplement: Supplementary file 1 — The online version contains supplementary material available at (DOCX 359 KB). [file 251_2025_1376_MOESM1_ESM.docx]

**Supplementary Information for:**

**Allele co-segregation and haplotype diversity of MHC IIβ genes in the small-spotted catshark *Scyliorhinus canicula***

Alessia Rota^1,2,3,4^, Ana Veríssimo^3,4^*, Arnaud Gaigher^3,5^*

^1^ Department of Earth and Environmental Sciences, University of Milano-Bicocca, Milan, Italy

^2^ National Biodiversity Future Centre (NBFC), Palermo, 90133, Italy

^3^ CIBIO‐InBIO, Research Center in Biodiversity and Genetic Resources, University of Porto, 4485-661 Vairão, Portugal

^4^ BIOPOLIS Program in Genomics, Biodiversity and Land Planning, CIBIO, Campus de Vairão, 4485-661 Vairão, Portugal

^5^ Research Unit for Evolutionary Immunogenomics, Department of Biology, University of Hamburg, Hamburg, Germany

*Co-corresponding authors: averissimo@cibio.up.pt; arnaud.gaigher@gmail.com

**Table S1**: Composition of the optimised primer mix of the forward and reverse primers used for the co-amplification of 11 microsatellites in *Scyliorhinus canicula* (Griffiths et al. 2011). All primers are labelled using fluorescent dyes as indicated.

|  |  | **Quantity (µl)** | |
| --- | --- | --- | --- |
| **Type** | **Name** | **Forward (10 µM)** | **Reverse (10 µM)** |
| Marker | Scan02 | 2.4 | 2.4 |
|  | Scan05 | 2.4 | 2.4 |
|  | Scan10 | 3.0 | 3.0 |
|  | Scan12 | 2.4 | 2.4 |
|  | Scan03 | 3.6 | 3.6 |
|  | Scan15 | 2.4 | 2.4 |
|  | Scan06 | 6.0 | 6.0 |
|  | Scan09 | 2.4 | 2.4 |
|  | Scan13 | 2.4 | 2.4 |
|  | Scan04 | 2.4 | 2.4 |
|  | Scan16 | 3.0 | 3.0 |
| Dye | NED | 7.8 | |
|  | FAM | 14.4 | |
|  | VIC | 10.2 | |
| H20 |  | 202.8 | |
|  | Final volume (µl) | 300 | |

**Table S2**: Nucleotide sequence, annealing temperature and fragment length of the primer combinations used for the amplification of MHC IIβ1 (exon 2) and β2 (exon 3) lineages in *Scyliorhinus canicula*. To amplify the MHC IIβ2 B lineage, an equal ratio of the two forward primers β2_B03F and β2_B52F has been used.

| **Target lineage** | **Forward primer ID** | **Forward sequence** | **Reverse primer ID** | **Reverse sequence** | **Ta** | **Amplicon size** |
| --- | --- | --- | --- | --- | --- | --- |
| β1 A/B | NF2 | TCTCACAGGGGCTCACA | NR2 | CCGCTCTCACCTYTCCGG | 58° | 279 bp |
| β1 C | DF2 | CTCTTCTAGGGGCTCATACC | DR2 | CCGCTCTCACCTTTCCTGG | 60° | 277 bp |
| β1 A | NF2 | TCTCACAGGGGCTCACA | β1_AR3 | GACAGCGCCGACTGTTCCATGA | 65° | 261 bp |
| β1 B | NF2 | TCTCACAGGGGCTCACA | β1_NR2B_11R | ATGCCAATGTTAGTCTGACA | 60° | 246 bp |
| β2 A | β2_AintF2 | GGAGGAGAGTTAGAAATGATG | β2_AintR1 | GATTTTGACAGACCTGCTCAG | 59° | 452 bp |
| β2 B | β2_B03F | ACTGGATTTTACCCTGCAA | classIIDBB_β2R | CAGGCTGMTGTGWKYCAC | 60° | 162 bp |
| β2 B | β2_B52F | ACTGGATTTTACCCTCCAG | classIIDBB_β2R | CAGGCTGMTGTGWKYCAC | 60° | 162 bp |
| β2 C | β2_CF | GAAACCAACAGTCACTATCCGC | β2_CR2 | CGACAGGTATATTTATCACCAG | 58.5° | 237 bp |

**Table S3**: Multilocus genotypes for 76 individuals of *Scyliorhinus canicula* at 11 microsatellite loci. Cells coded with “0” refer to loci with no data.

| **Sample ID** | **Scan02** | | **Scan03** | | **Scan04** | | **Scan05** | | **Scan06_2** | | **Scan09** | | **Scan10** | | **Scan12** | | **Scan13** | | **Scan15** | | **Scan16_2** | |
| --- | --- | --- | --- | --- | --- | --- | --- | --- | --- | --- | --- | --- | --- | --- | --- | --- | --- | --- | --- | --- | --- | --- |
| Group1_F1 | 134 | 138 | 186 | 192 | 255 | 255 | 198 | 200 | 229 | 229 | 132 | 132 | 270 | 276 | 119 | 119 | 203 | 203 | 254 | 254 | 283 | 283 |
| Group1_F10 | 136 | 138 | 188 | 192 | 255 | 255 | 198 | 200 | 229 | 229 | 132 | 132 | 270 | 276 | 119 | 127 | 203 | 205 | 254 | 254 | 283 | 283 |
| Group1_F11 | 0 | 0 | 0 | 0 | 0 | 0 | 0 | 0 | 0 | 0 | 0 | 0 | 0 | 0 | 0 | 0 | 0 | 0 | 0 | 0 | 0 | 0 |
| Group1_F13 | 138 | 138 | 188 | 188 | 255 | 255 | 198 | 200 | 229 | 233 | 132 | 132 | 270 | 276 | 119 | 127 | 203 | 203 | 254 | 256 | 283 | 283 |
| Group1_F15 | 138 | 140 | 188 | 188 | 255 | 255 | 198 | 200 | 229 | 239 | 132 | 132 | 266 | 270 | 117 | 119 | 203 | 205 | 252 | 256 | 283 | 283 |
| Group1_F16 | 138 | 138 | 186 | 192 | 255 | 255 | 198 | 200 | 229 | 233 | 132 | 132 | 270 | 276 | 119 | 127 | 203 | 203 | 252 | 254 | 283 | 283 |
| Group1_F17 | 134 | 136 | 188 | 192 | 255 | 255 | 198 | 200 | 229 | 229 | 132 | 132 | 270 | 276 | 119 | 127 | 203 | 203 | 252 | 256 | 283 | 283 |
| Group1_F18 | 134 | 136 | 188 | 192 | 255 | 255 | 198 | 200 | 229 | 233 | 132 | 132 | 270 | 276 | 119 | 127 | 203 | 205 | 254 | 256 | 283 | 283 |
| Group1_F19 | 134 | 138 | 188 | 192 | 255 | 255 | 198 | 200 | 229 | 229 | 132 | 132 | 270 | 276 | 119 | 119 | 203 | 205 | 252 | 256 | 283 | 283 |
| Group1_F2 | 134 | 138 | 188 | 188 | 255 | 255 | 198 | 200 | 229 | 229 | 132 | 132 | 270 | 276 | 119 | 127 | 203 | 203 | 254 | 256 | 283 | 283 |
| Group1_F20 | 134 | 138 | 186 | 188 | 255 | 263 | 198 | 200 | 229 | 235 | 132 | 134 | 270 | 270 | 117 | 119 | 203 | 203 | 254 | 254 | 275 | 283 |
| Group1_F21 | 134 | 138 | 188 | 188 | 255 | 255 | 198 | 200 | 229 | 229 | 132 | 132 | 266 | 270 | 117 | 119 | 203 | 203 | 252 | 254 | 283 | 283 |
| Group1_F22 | 134 | 136 | 186 | 186 | 255 | 255 | 198 | 200 | 229 | 229 | 132 | 132 | 270 | 270 | 117 | 119 | 203 | 203 | 252 | 256 | 283 | 283 |
| Group1_F23 | 136 | 136 | 186 | 186 | 255 | 255 | 198 | 200 | 229 | 237 | 132 | 132 | 270 | 270 | 119 | 119 | 203 | 205 | 256 | 256 | 275 | 283 |
| Group1_F3 | 136 | 138 | 186 | 188 | 255 | 255 | 198 | 200 | 229 | 229 | 132 | 132 | 270 | 276 | 119 | 119 | 203 | 205 | 254 | 254 | 283 | 283 |
| Group1_F4 | 136 | 140 | 186 | 188 | 255 | 255 | 198 | 200 | 229 | 241 | 132 | 132 | 270 | 270 | 115 | 119 | 203 | 205 | 254 | 258 | 283 | 283 |
| Group1_F5 | 138 | 140 | 186 | 188 | 255 | 255 | 198 | 200 | 229 | 237 | 132 | 132 | 270 | 270 | 119 | 119 | 203 | 205 | 254 | 256 | 275 | 283 |
| Group1_F6 | 134 | 136 | 186 | 192 | 255 | 255 | 198 | 200 | 229 | 233 | 132 | 132 | 270 | 276 | 119 | 127 | 203 | 203 | 252 | 254 | 283 | 283 |
| Group1_F7 | 136 | 138 | 186 | 188 | 255 | 265 | 198 | 200 | 229 | 241 | 132 | 132 | 270 | 270 | 115 | 119 | 203 | 203 | 254 | 258 | 283 | 283 |
| Group1_F8 | 136 | 138 | 186 | 192 | 255 | 255 | 198 | 200 | 229 | 229 | 132 | 132 | 270 | 276 | 119 | 119 | 203 | 203 | 254 | 254 | 283 | 283 |
| Group1_F9 | 136 | 136 | 186 | 186 | 255 | 265 | 198 | 200 | 229 | 237 | 132 | 132 | 270 | 270 | 115 | 119 | 203 | 205 | 256 | 258 | 283 | 283 |
| Group1_mother1 | 136 | 138 | 186 | 188 | 255 | 255 | 200 | 200 | 229 | 229 | 132 | 132 | 270 | 270 | 119 | 119 | 203 | 203 | 254 | 256 | 283 | 283 |
| Group2_father1 | 134 | 140 | 186 | 192 | 263 | 265 | 198 | 198 | 233 | 235 | 132 | 132 | 274 | 274 | 119 | 121 | 203 | 203 | 254 | 254 | 285 | 285 |
| Group2_father2 | 134 | 134 | 194 | 194 | 255 | 255 | 198 | 198 | 229 | 233 | 132 | 132 | 270 | 276 | 119 | 121 | 203 | 203 | 252 | 258 | 283 | 283 |
| Group2_father3 | 132 | 142 | 186 | 194 | 255 | 255 | 198 | 200 | 229 | 241 | 132 | 132 | 270 | 274 | 117 | 119 | 195 | 203 | 256 | 258 | 279 | 283 |
| Group2_mother1 | 134 | 134 | 186 | 186 | 255 | 263 | 198 | 200 | 229 | 229 | 132 | 132 | 270 | 274 | 117 | 121 | 203 | 203 | 256 | 258 | 283 | 285 |
| Group2_mother2 | 132 | 134 | 186 | 194 | 263 | 263 | 198 | 198 | 233 | 235 | 132 | 132 | 274 | 274 | 121 | 121 | 203 | 203 | 254 | 256 | 285 | 285 |
| Group2_mother3 | 134 | 140 | 192 | 194 | 255 | 263 | 198 | 198 | 233 | 237 | 134 | 134 | 274 | 274 | 121 | 123 | 203 | 203 | 254 | 256 | 283 | 285 |
| Group2_mother4 | 136 | 144 | 186 | 194 | 255 | 255 | 198 | 200 | 229 | 239 | 132 | 132 | 270 | 274 | 119 | 121 | 203 | 205 | 252 | 256 | 279 | 283 |
| Group2_pup1A2 | 134 | 142 | 186 | 186 | 255 | 255 | 198 | 198 | 229 | 229 | 132 | 132 | 270 | 270 | 117 | 117 | 195 | 203 | 256 | 258 | 283 | 285 |
| Group2_pup1A3 | 132 | 136 | 186 | 186 | 255 | 255 | 198 | 198 | 229 | 229 | 132 | 132 | 270 | 274 | 119 | 121 | 195 | 203 | 256 | 258 | 279 | 283 |
| Group2_pup1A4 | 134 | 142 | 186 | 186 | 255 | 255 | 198 | 200 | 229 | 229 | 132 | 132 | 270 | 274 | 119 | 121 | 203 | 203 | 258 | 258 | 283 | 285 |
| Group2_pup1A6 | 134 | 138 | 192 | 194 | 255 | 265 | 198 | 198 | 233 | 237 | 132 | 132 | 274 | 274 | 121 | 121 | 203 | 203 | 254 | 254 | 283 | 285 |
| Group2_pup1B2 | 136 | 140 | 186 | 194 | 255 | 265 | 198 | 198 | 229 | 235 | 132 | 132 | 274 | 274 | 119 | 119 | 203 | 203 | 254 | 256 | 283 | 285 |
| Group2_pup1B4 | 132 | 134 | 186 | 186 | 255 | 263 | 198 | 198 | 229 | 241 | 132 | 132 | 270 | 274 | 119 | 121 | 203 | 203 | 256 | 256 | 279 | 283 |
| **Sample ID** | **Scan02** | | **Scan03** | | **Scan04** | | **Scan05** | | **Scan06_2** | | **Scan09** | | **Scan10** | | **Scan12** | | **Scan13** | | **Scan15** | | **Scan16_2** | |
| Group2_pup1B6 | 132 | 144 | 186 | 194 | 255 | 255 | 198 | 198 | 239 | 241 | 132 | 132 | 270 | 270 | 117 | 119 | 203 | 203 | 252 | 256 | 279 | 279 |
| Group2_pup1C3 | 134 | 144 | 194 | 194 | 255 | 255 | 198 | 198 | 233 | 239 | 132 | 132 | 270 | 274 | 121 | 121 | 203 | 203 | 252 | 258 | 279 | 283 |
| Group2_pup1C4 | 134 | 144 | 186 | 186 | 255 | 265 | 198 | 198 | 229 | 233 | 132 | 132 | 270 | 274 | 119 | 119 | 203 | 205 | 254 | 256 | 283 | 285 |
| Group2_pup1C6 | 134 | 138 | 192 | 192 | 263 | 263 | 198 | 198 | 233 | 233 | 132 | 132 | 274 | 274 | 119 | 119 | 203 | 203 | 254 | 256 | 285 | 285 |
| Group2_pup1D2 | 134 | 140 | 186 | 192 | 263 | 265 | 198 | 200 | 229 | 235 | 132 | 132 | 270 | 274 | 119 | 121 | 203 | 203 | 254 | 258 | 283 | 285 |
| Group2_pup1D6 | 134 | 140 | 186 | 186 | 255 | 265 | 198 | 200 | 229 | 233 | 132 | 132 | 274 | 274 | 119 | 121 | 203 | 203 | 254 | 258 | 283 | 285 |
| Group2_pup1E2 | 134 | 134 | 186 | 186 | 255 | 263 | 198 | 198 | 229 | 233 | 132 | 132 | 274 | 274 | 117 | 121 | 203 | 203 | 254 | 256 | 285 | 285 |
| Group2_pup1E3 | 134 | 142 | 194 | 194 | 255 | 255 | 198 | 200 | 229 | 229 | 132 | 132 | 274 | 274 | 117 | 119 | 203 | 203 | 258 | 258 | 283 | 283 |
| Group2_pup1E5 | 134 | 142 | 186 | 186 | 255 | 263 | 200 | 200 | 229 | 241 | 132 | 132 | 274 | 274 | 117 | 119 | 195 | 203 | 258 | 258 | 279 | 285 |
| Group2_pup1E6 | 132 | 140 | 186 | 194 | 255 | 265 | 198 | 198 | 235 | 237 | 132 | 132 | 274 | 274 | 119 | 121 | 203 | 203 | 254 | 254 | 283 | 285 |
| Group2_pup1F3 | 134 | 134 | 192 | 192 | 255 | 265 | 198 | 198 | 229 | 233 | 132 | 132 | 274 | 274 | 117 | 121 | 203 | 203 | 254 | 258 | 283 | 285 |
| Group2_pup1F5 | 134 | 134 | 192 | 192 | 255 | 265 | 198 | 198 | 229 | 233 | 132 | 132 | 274 | 274 | 117 | 121 | 203 | 203 | 254 | 258 | 283 | 285 |
| Group2_pup1G10 | 132 | 140 | 186 | 194 | 255 | 265 | 198 | 198 | 235 | 237 | 132 | 132 | 274 | 274 | 119 | 121 | 203 | 203 | 254 | 254 | 283 | 285 |
| Group2_pup1G2 | 134 | 144 | 186 | 194 | 255 | 263 | 198 | 200 | 229 | 235 | 132 | 132 | 270 | 274 | 119 | 121 | 203 | 205 | 252 | 254 | 279 | 285 |
| Group2_pup1G5 | 134 | 142 | 186 | 186 | 255 | 255 | 200 | 200 | 229 | 241 | 132 | 132 | 270 | 274 | 117 | 117 | 195 | 203 | 256 | 258 | 283 | 283 |
| Group2_pup1H1 | 134 | 142 | 186 | 186 | 255 | 263 | 198 | 200 | 229 | 229 | 132 | 132 | 274 | 274 | 117 | 121 | 203 | 203 | 256 | 258 | 283 | 283 |
| Group2_pup1H10 | 138 | 140 | 186 | 186 | 255 | 263 | 198 | 198 | 233 | 237 | 132 | 132 | 274 | 274 | 121 | 121 | 203 | 203 | 254 | 254 | 283 | 285 |
| Group2_pup1H5 | 134 | 144 | 186 | 192 | 255 | 263 | 198 | 198 | 233 | 239 | 132 | 132 | 270 | 274 | 119 | 119 | 203 | 205 | 252 | 254 | 283 | 285 |
| Group2_pup1I1 | 134 | 142 | 186 | 186 | 255 | 263 | 198 | 200 | 229 | 229 | 132 | 132 | 270 | 270 | 117 | 121 | 203 | 203 | 258 | 258 | 279 | 283 |
| Group2_pup1I10 | 132 | 144 | 186 | 186 | 255 | 255 | 198 | 200 | 229 | 239 | 132 | 132 | 270 | 274 | 119 | 119 | 203 | 205 | 252 | 258 | 279 | 283 |
| Group2_pup1I3 | 134 | 140 | 186 | 186 | 263 | 265 | 198 | 198 | 229 | 233 | 132 | 132 | 274 | 274 | 119 | 121 | 203 | 203 | 254 | 258 | 283 | 285 |
| Group2_pup1I5 | 134 | 140 | 186 | 186 | 263 | 265 | 198 | 200 | 229 | 233 | 132 | 132 | 274 | 274 | 117 | 121 | 203 | 203 | 254 | 256 | 283 | 285 |
| Group2_pup1I6 | 134 | 140 | 186 | 192 | 263 | 265 | 198 | 200 | 229 | 233 | 132 | 132 | 274 | 274 | 117 | 119 | 203 | 203 | 254 | 258 | 285 | 285 |
| Group2_pup1J1 | 136 | 142 | 194 | 194 | 255 | 255 | 200 | 200 | 229 | 239 | 132 | 132 | 270 | 270 | 119 | 121 | 203 | 205 | 252 | 258 | 279 | 283 |
| Group2_pup1J10 | 134 | 142 | 186 | 186 | 255 | 255 | 198 | 200 | 229 | 241 | 132 | 132 | 274 | 274 | 117 | 121 | 203 | 203 | 256 | 256 | 279 | 285 |
| Group2_pup1J3 | 134 | 136 | 186 | 192 | 255 | 265 | 198 | 198 | 229 | 235 | 132 | 132 | 270 | 274 | 121 | 121 | 203 | 205 | 254 | 256 | 283 | 285 |
| Group2_pup1J5 | 138 | 140 | 192 | 192 | 255 | 265 | 198 | 198 | 235 | 237 | 132 | 132 | 274 | 274 | 121 | 121 | 203 | 203 | 254 | 254 | 283 | 285 |
| Group2_pup2A6 | 134 | 134 | 194 | 194 | 255 | 263 | 198 | 200 | 229 | 229 | 132 | 132 | 270 | 276 | 117 | 121 | 203 | 203 | 258 | 258 | 283 | 285 |
| Group2_pup2A9 | 134 | 144 | 186 | 194 | 255 | 263 | 198 | 200 | 229 | 235 | 132 | 132 | 270 | 274 | 119 | 121 | 203 | 205 | 254 | 256 | 279 | 285 |
| Group2_pup2B1 | 132 | 140 | 192 | 192 | 255 | 265 | 198 | 198 | 235 | 237 | 132 | 132 | 274 | 274 | 119 | 121 | 203 | 203 | 254 | 254 | 283 | 285 |
| Group2_pup2B2 | 134 | 134 | 0 | 0 | 265 | 265 | 198 | 198 | 229 | 233 | 132 | 134 | 274 | 274 | 117 | 121 | 203 | 203 | 254 | 254 | 0 | 0 |
| Group2_pup2B9 | 134 | 134 | 0 | 0 | 255 | 263 | 198 | 200 | 229 | 229 | 132 | 132 | 270 | 270 | 121 | 121 | 203 | 203 | 258 | 258 | 283 | 285 |
| Group2_pup2C1 | 132 | 136 | 186 | 186 | 255 | 255 | 200 | 200 | 229 | 239 | 132 | 132 | 270 | 274 | 117 | 119 | 195 | 203 | 256 | 256 | 279 | 279 |
| Group2_pup2C2 | 134 | 134 | 192 | 192 | 263 | 265 | 198 | 198 | 229 | 233 | 132 | 132 | 274 | 274 | 119 | 121 | 203 | 203 | 256 | 256 | 283 | 285 |
| Group2_pup2C9 | 134 | 134 | 186 | 194 | 255 | 263 | 198 | 198 | 229 | 233 | 132 | 132 | 274 | 276 | 117 | 121 | 203 | 203 | 258 | 258 | 283 | 285 |
| Group2_pup2D1 | 134 | 134 | 194 | 194 | 255 | 255 | 198 | 200 | 229 | 229 | 132 | 132 | 270 | 274 | 121 | 121 | 203 | 203 | 252 | 258 | 283 | 283 |
| Group2_pup2D2 | 134 | 134 | 186 | 186 | 255 | 263 | 198 | 200 | 229 | 233 | 132 | 132 | 270 | 276 | 117 | 119 | 203 | 203 | 252 | 256 | 283 | 283 |
| Group2_pup2D9 | 136 | 140 | 186 | 186 | 255 | 265 | 198 | 198 | 229 | 233 | 132 | 132 | 270 | 274 | 119 | 119 | 203 | 205 | 254 | 256 | 283 | 285 |
| Group2_pup2E1 | 134 | 134 | 194 | 194 | 255 | 255 | 198 | 200 | 229 | 233 | 132 | 132 | 274 | 276 | 119 | 121 | 203 | 203 | 256 | 258 | 283 | 283 |
| Group2_pup2G1 | 134 | 144 | 192 | 194 | 255 | 265 | 198 | 200 | 235 | 239 | 132 | 132 | 270 | 274 | 119 | 121 | 203 | 205 | 254 | 256 | 283 | 285 |
| Group2_pup2I1 | 134 | 134 | 192 | 192 | 255 | 265 | 198 | 200 | 229 | 235 | 132 | 132 | 270 | 274 | 119 | 121 | 203 | 203 | 254 | 258 | 285 | 285 |

**Table S4**: Results of the Cervus reconstruction showing the most likely mother and father for each offspring in group 2. Mother (M) or Father (F) are highlighted red in cases where the respective LOD score is < 0.

| **Offspring ID** | **Candidate mother** | **Candidate father** |
| --- | --- | --- |
| 1A2 | M1 | F3 |
| 1A3 | M4, M1 | F3 |
| 1A4 | M1 | F3, F2 |
| 1A6 |  |  |
| 1B2 | M4 | F1 |
| 1B4 | M1, M2 | F3 |
| 1B6 | M4 | F3 |
| 1C3 | M4 | F2 |
| 1C4 | M4 |  |
| 1C6 |  | F1 |
| 1D2 | M1 | F1 |
| 1D6 | M1 | F1 |
| 1E2 | M1, M2 | F1 |
| 1E3 | M1, M4 | F3, F2 |
| 1E5 | M1 | F3 |
| 1E6 |  | F1 |
| 1F3 | M1 | F1 |
| 1F5 | M1 | F1 |
| 1G10 |  | F1 |
| 1G2 | M4 | F2 |
| 1G5 | M1, M4 | F3 |
| 1H1 | M1, M2 | F3 |
| 1H10 |  | F1 |
| 1H5 | M4 | F2 |
| 1I1 | M1 | F3, F2 |
| 1I10 | M4 | F3 |
| 1I3 | M2, M1 | F1 |
| 1I5 | M1 | F1 |
| 1I6 | M1 | F1 |
| 1J1 | M4 | F3 |
| 1J10 | M1, M2, M4 | F3 |
| 1J3 | M4 |  |
| 1J5 | M3 | F1 |
| 2A6 | M1 | F2, F3 |
| 2A9 | M4, M2 |  |
| 2B1 |  | F1 |
| 2B2 |  |  |
| 2B9 | M1 | F2 |
| 2C1 | M4 | F3 |
| 2C2 | M1 | F1 |
| 2C9 | M1, M2 | F2, F1 |
| 2D1 | M1, M4 | F2 |
| 2D2 | M1 | F2 |
| 2D9 | M4 |  |
| 2E1 | M4, M1, M3 | F2 |
| 2G1 | M4 |  |
| 2I1 | M1 | F1 |

**Table S5**: Inference of genetic linkage between MHC IIβ lineages of the small-spotted catshark using family data. (a) Family #1, (b) Family #2, (c) Family #3, (d) Family #4, and (e) Family #5. Only Family #1 is part of Group 1. Family #1 includes several putative fathers. Haplotypes with * indicate that they were not considered for detecting recombination events (see Table 3).

**Table S6:** Number of alleles from lineages A, B and C, and number of lineages present per individual of *S. canicula* sampled from the same population, taken from Gaigher et al. (2023).

| Individual Ref. | No. of A alleles | No. of B alleles | No. of C alleles | No. of lineages |
| --- | --- | --- | --- | --- |
| ALG_709 | 1 | 2 | 1 | 3 |
| ALG_710 | 0 | 2 | 1 | 2 |
| ALG_712 | 0 | 3 | 2 | 2 |
| ALG_714 | 0 | 3 | 1 | 2 |
| ALG_718 | 0 | 2 | 2 | 2 |
| ALG_719 | 1 | 3 | 2 | 3 |
| ALG_722 | 1 | 2 | 1 | 3 |
| ALG_723 | 0 | 3 | 2 | 2 |
| ALG_725 | 2 | 1 | 1 | 3 |
| ALG_726 | 0 | 3 | 1 | 2 |
| ALG_727 | 0 | 2 | 2 | 2 |
| ALG_728 | 1 | 2 | 1 | 3 |
| ALG_732 | 1 | 1 | 2 | 3 |
| ALG_733 | 0 | 2 | 2 | 2 |
| ALG_740 | 0 | 4 | 2 | 2 |
| ALG_741 | 0 | 3 | 1 | 2 |
| ALG_744 | 0 | 2 | 1 | 2 |
| ALG_745 | 1 | 1 | 2 | 3 |
| ALG_749 | 0 | 2 | 2 | 2 |
| ALG_750 | 0 | 2 | 1 | 2 |
| ALG_751 | 1 | 2 | 1 | 3 |
| ALG_757 | 0 | 3 | 1 | 2 |
| PTM734 | 0 | 2 | 1 | 2 |
| PTM735 | 1 | 2 | 1 | 3 |
| PTM746 | 1 | 1 | 1 | 3 |

**Figure S1**: Graphic illustration of the primers' location for the amplification of MHC IIβ1 (exon 2) and β2 (exon 3) in *Scyliorhinus canicula*. The different colours identify the primer pair combinations (see details in Table S2). Primers placed above the exon amplify for gene C, while primers below the exon amplify for lineage A and/or lineage B.


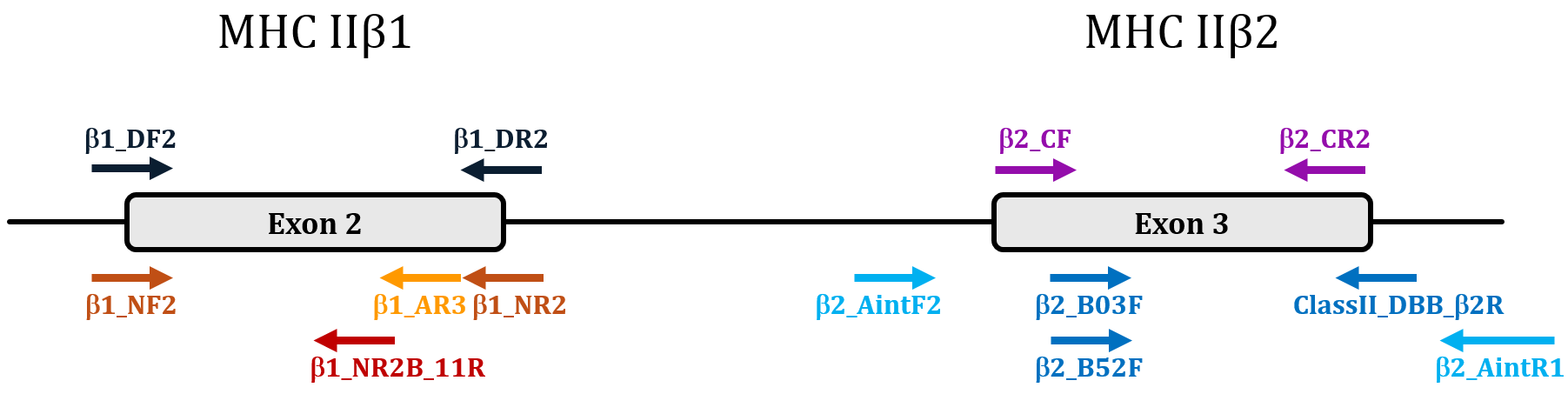


**Figure S2**: Phylogenetic network of small-spotted catshark MHC IIβ exon 2 alleles. Black dots represent the sequences extracted from the *S. canicula* reference genome sScyCan1.1 (BioProject PRJEB35945). Grey dots represent the new sequences detected in this study (GenBank accession numbers: PP982215-PP982223). Other exon 2 allele sequences were obtained from Gaigher et al. (2023).


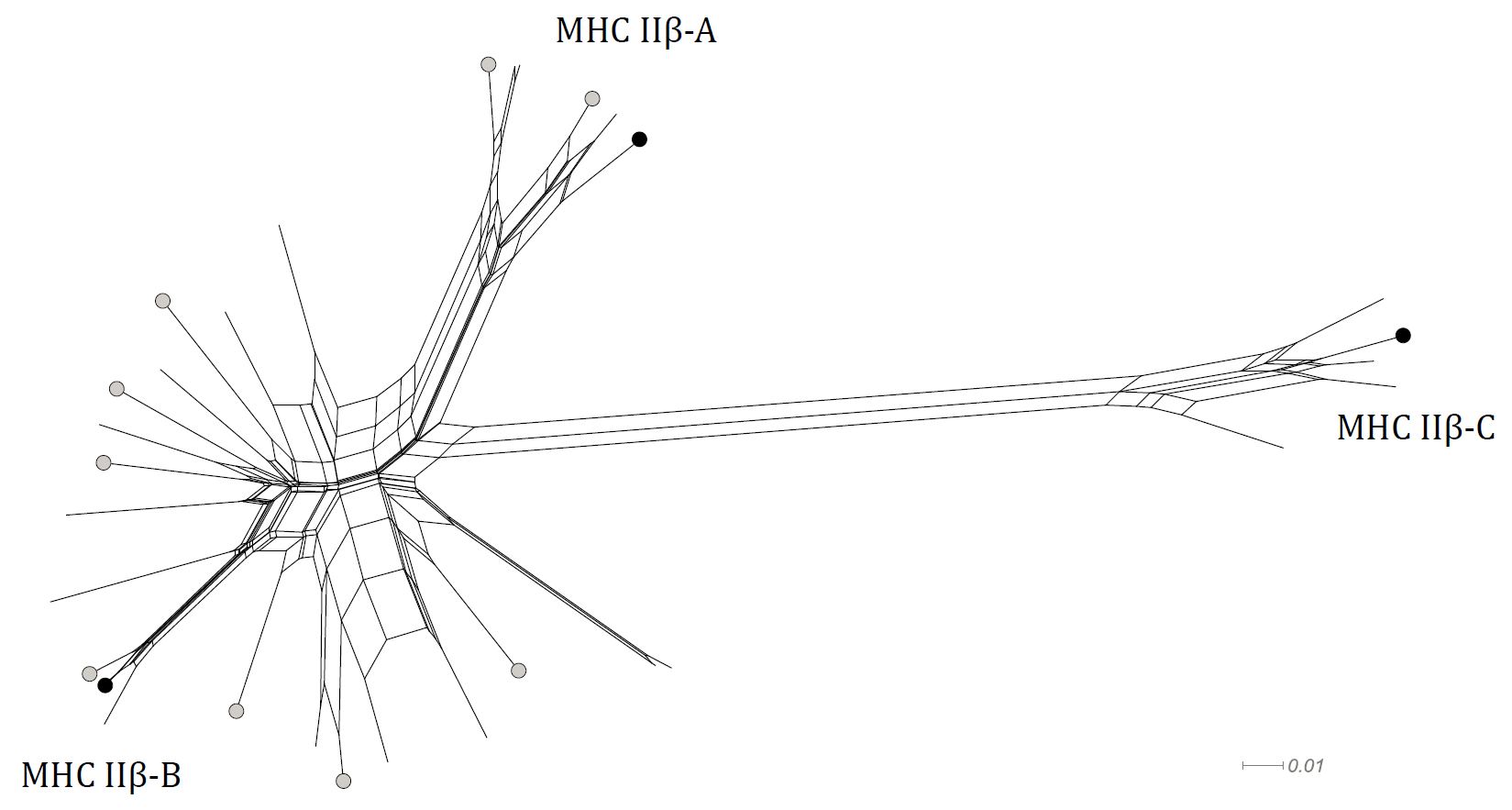


**
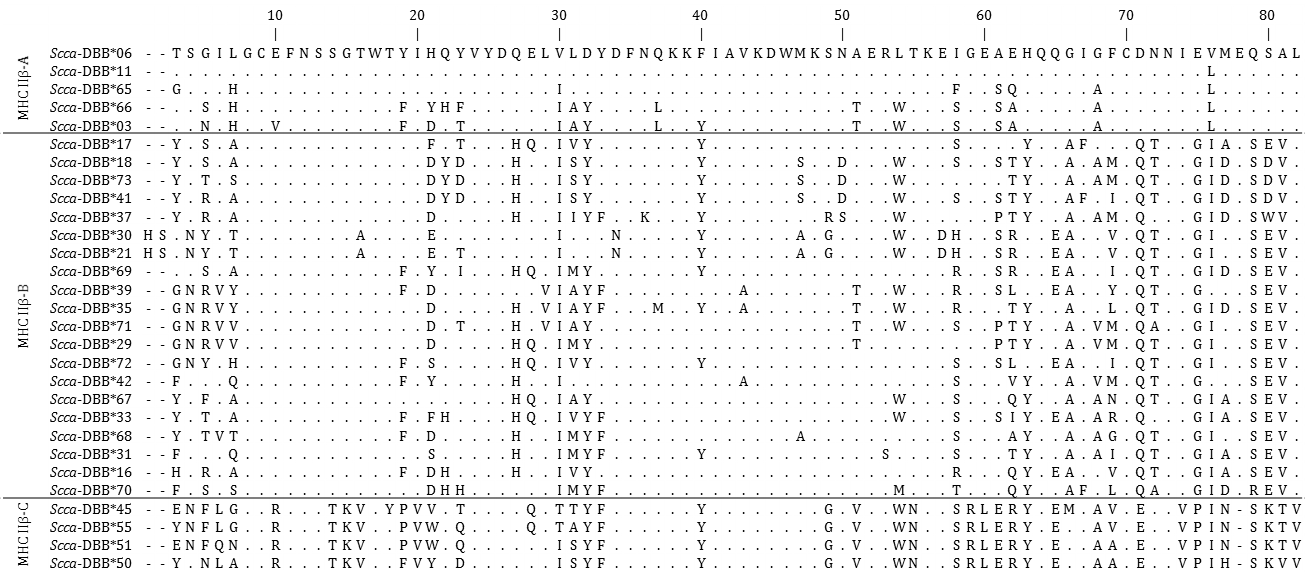
 Figure S3**: Amino acid sequences of MHC IIβ exon 2 alleles detected in this study.

**Figure S4:** Alignment of transcripts referring to loci representing MHC IIβ lineages A, B and C in *Scyliorhinus canicula* (from the gene annotation of the sScyCan1.1 genome assembly). Untranslanted regions in the 5´and 3´ends of the coding sequence are highlighted in dark grey and light grey, respectively. Dots refer to nucleotide agreements to the reference sequence on top; dashes refer to alignment gaps.

**Figure S5:** Comparison of the number of alleles per lineage per individual in a sample of 25 unrelated individuals of *S. canicula* from Gaigher et al. (2023). Number of alleles for a) lineages A and C, b) lineages B and C, and c) lineages A and B. Size of circles is proportional to number of individuals with the combination of allele numbers per lineage.
